# Prevalence of subclinical lung cancer detected at autopsy: a systematic review

**DOI:** 10.1186/s12885-023-11224-3

**Published:** 2023-08-24

**Authors:** Asha Bonney, Kayo Togawa, Michelle Ng, Michael Christie, Kwun M Fong, Henry Marshall, Katharine See, Cameron Patrick, Daniel Steinfort, Renee Manser

**Affiliations:** 1 Department of Respiratory and Sleep Medicine, The Royal Melbourne Hospital, 300 Grattan Street, Parkville, VIC Australia; 2 Department of Medicine, The University of Melbourne, Melbourne, Australia; 3 Division of Surveillance and Policy Evaluation, National Cancer Center Institute for Cancer Control, Tokyo, Japan; 4 Cardiac Surgery Department, Austin Hospital, Heidelberg, Australia; 5 Department of Anatomical Pathology, The Royal Melbourne Hospital, Melbourne, Australia; 6 Thoracic Medicine Program, The Prince Charles Hospital, Chermside, Australia; 7 UQ Thoracic Research Centre, School of Medicine, The University of Queensland, Brisbane, Australia; 8 Department of Respiratory Medicine, Northern Hospital, Epping, Australia; 9 Statistical Consulting Centre, School of Mathematics and Statistics, The University of Melbourne, Melbourne, Australia

**Keywords:** Lung cancer, Subclinical, Latent, Screening, Overdiagnosis, Autopsy

## Abstract

**Background:**

Lung cancer screening in high-risk populations with low-dose computed tomography is supported by international associations and recommendations. Overdiagnosis is considered a risk of screening with associated harms. The aim of this paper is to determine the prevalence of subclinical lung cancer diagnosed post-mortem to better understand the reservoir of subclinical lung cancer.

**Methods:**

We searched EMBASE, PubMed, and MEDLINE databases from inception until March 2022 with no language restrictions. We considered all studies with ≥100 autopsies in adults. Two reviewers independently assessed eligibility of studies, extracted data, and assessed risk of bias of included studies. We performed a meta-analysis using a random-effects model for prevalence of subclinical lung cancer diagnosed post-mortem with sensitivity and subgroup analyses.

**Results:**

A total of 13 studies with 16 730 autopsies were included. Pooled prevalence was 0.4% (95% CI 0.20 to 0.82%, I^2^ = 84%, tau^2^ = 1.19, low certainty evidence,16 730 autopsies). We performed a sensitivity analysis excluding studies which did not specify exclusion of children in their cohort, with a pooled prevalence of subclinical lung cancer of 0.87% (95% CI 0.48 to 1.57%, I^2^ = 71%, tau^2^ = 0.38, 6998 autopsies, 8 studies).

**Conclusions:**

This is the first published systematic review to evaluate the prevalence of post-mortem subclinical lung cancer. Compared to autopsy systematic reviews in breast, prostate and thyroid cancers, the pooled prevalence is lower in lung cancer for subclinical cancer. This result should be interpreted with caution due to the included studies risk of bias and heterogeneity, with further high-quality studies required in target screening populations.

**Supplementary Information:**

The online version contains supplementary material available at 10.1186/s12885-023-11224-3.

## Background

Lung cancer is the second most diagnosed cancer in the world and remains the leading cause of cancer-related death, responsible for almost 1.8 million deaths globally in 2020 [1]. This is despite lung cancer incidence rates declining in males in Australia, Canada, Denmark, Germany, Netherlands, New Zealand and United States of America (USA), although concerningly lung cancer incidence rates in younger women (30 to 49 years old) are trending upwards [2, 3].

A recent systematic review of randomised controlled trials (RCTs) using low-dose computed tomography (LDCT) for lung cancer screening in high-risk populations (current or former smokers) concluded a reduction in lung cancer-related mortality of 21% compared with control groups (no screening or chest radiograph screening), (relative risk (RR) 0.79, 95% confidence interval (CI) 0.72 to 0.87) [4]. Additionally, there was also a reduction in all-cause mortality of 5% (RR 0.95, 95% CI 0.91 to 0.99) [4]. Multiple international guidelines now recommend screening for lung cancer in high-risk smoking populations with LDCT, with South Korea implementing a national screening program [5] and the USA funding screening in individuals meeting criteria (current or former smokers with ≥20 pack-year history, quit ≤15 years ago, and aged between 50 and 80 years old) [6].

Whilst there are limited data on the harms of LDCT screening, one significant consideration is the risk of overdiagnosis. Overdiagnosis refers to detection and diagnosis of lung cancer that would never have caused the patient harm or death [7]. In lung cancer, there remains some uncertainty about the extent of overdiagnosis, with the meta-analysis of lung cancer screening in high-risk groups with LDCT RCTs reporting an estimated range of 0 to 36% of lung cancers being overdiagnosed at 10 or more years [4]. This estimate was graded low certainty evidence due to the quality of the studies and heterogeneity. Overdiagnosis of lung cancer can cause harm by resulting in unnecessary investigations and treatment (most commonly surgery in early-stage disease), along with associated complications and cost.

There are many different histological subtypes of lung cancer as defined by the World Health Organization (WHO) [8]. Of note, in the systematic review of LDCT lung cancer screening RCTs in high risk populations, the longer-term results (≥ 7 years post-randomisation), demonstrated probably no difference in the prevalence of small cell lung cancer (SCLC) and squamous cell carcinoma between the groups who received LDCT screening and those who did not [4]. Conversely, adenocarcinoma was more prevalent in the LDCT screening group compared to the control group. This is potentially related to the more variable volume doubling time (VDT) of adenocarcinomas, with the micropapillary subtype having median VDT of 229 days and lepidic adenocarcinomas having a median VDT of 647 days in one study [9].

Systematic reviews of cancers diagnosed at autopsy have already contributed immensely to understanding the risk of overdiagnosis and reservoir of subclinical cancer in prostate, breast, and thyroid cancers [10–12]. In prostate cancer, there was an increased prevalence of subclinical prostate cancer with increasing age, with the estimated mean adjusted prevalence of prostate cancer diagnosed at autopsy in men aged > 79 years old of 59% (95% CI 48–71%) [11]. In breast cancer, the estimated mean prevalence diagnosed at autopsy was 20% (including precursor lesions) [10]. For thyroid cancer diagnosed at autopsy, the pooled prevalence was 11% (95% CI 6 to 16%) [12]. The significant risk of overdiagnosis in prostate and thyroid cancer has contributed to recommendations against routine screening in asymptomatic individuals [13, 14]. Whilst breast cancer screening is recommended routinely in many countries, overdiagnosis is a recognised risk and consideration when counselling women [15].

There has been no previously published systematic review of the prevalence of subclinical lung cancer diagnosed at autopsy. However, in one Swedish autopsy study of 7020 adults with a mean age of 55 years for men and 58 years for women, only 5 had a post-mortem diagnosis of subclinical lung cancer (0.07% prevalence) [16]. This review is not only useful in the discussion around lung cancer screening, but also to explore potential associations between demographics and subgroups regarding possible predictors for subclinical lung cancer.

This review aims to describe the prevalence (or reservoir) of subclinical lung cancer detected at autopsy in adults.

## Methods

The systematic review was registered with the International Prospective Register of Systematic Reviews (PROSPERO registration: CRD42020140747) and reporting has been guided by the Preferred Reporting Items for Systematic Reviews and Meta-analyses (PRISMA) checklist [17].

### Criteria for considering studies for this review

We considered all studies with more than 100 autopsies in adults (aged 18 or over) who were not known to have lung cancer ante-mortem. We included studies which specified information about whether lung cancer caused or contributed to death or was subclinical.

### Search methods for identification of studies

We searched EMBASE, PubMed, and MEDLINE databases from inception until March 2022 using the following search strategy.

1. exp Lung Neoplasms/.
2. (lung* adj3 (neoplasm* or neoplasia* or cancer* or carcinoma* or adenocarcinoma* or tumour* or tumor* or malignan* or pre-malignan* or premalignan*)).tw.
3. Autopsy/.
4. (autops* or post-mortem* or post mortem*).tw.
5. (#1 or #2) AND (#3 or#4).
6. limit 5 to humans.

At least two review authors (AB, MN, KS, KT) independently screened all titles and abstracts retrieved by electronic searches using Covidence [18]. At least two review authors (AB, MN, KT) then obtained the full texts for all relevant studies and independently checked eligibility of each study against review eligibility criteria. We resolved discordant evaluations by discussion to reach consensus, and when necessary, involved a third review author (RM). We report the search results and study selection process using a PRISMA flow diagram [17].

The review authors (AB, RM) developed a data extraction form of which parts were adapted from the Checklist for Prevalence Studies [19]. Two review authors (AB and KT) independently extracted relevant data and performed a cross-check. If required, a third review author (RM) was consulted to reach consensus. We were not blinded to publication details. When there were multiple publications related to the same study, we chose the publication with the primary outcome as the study identifier. When data was missing or unsuitable for analysis, we (AB) contacted study authors to request further information using email addresses from study reports or registers where available.

We collected the following data.

- Source: citation, contact details.
- Eligibility criteria and reasons for exclusion.
- Methods: study design, total duration of study, number of centres and locations, autopsy rate.
- Characteristics of participants: number of participants, demographics (age, sex, exposures, lung cancer risk factors, co-morbidities).
- Autopsy: methodology.
- Results: lung cancer diagnoses, histology, stage of lung cancer, cause of death.
- Miscellaneous: funding source, conflicts of interest.

There were no validated tools for assessing the quality of autopsy studies for prevalence of subclinical cancer. The authors (AB and RM) developed a risk of bias (RoB) tool with components adapted from previous tools used in prevalence and diagnostic accuracy studies [20, 21]. Two review authors (AB and KT) independently applied the RoB tool to assess quality of included studies. We rated each domain of the tool as having ‘low’, ‘high’, or ‘unclear risk of bias for each study and supported the rating of each domain with a brief description. A third review author (RM) was consulted if required to reach consensus. We considered the following domains.

- Selection bias -patient selection: we scored ‘low risk’ when consecutive autopsy cases were included and there was avoidance of inappropriate exclusions, ‘high risk’ when non-consecutive autopsy cases were enrolled and/or there were inappropriate exclusions, and ‘unclear risk’ when there was insufficient information to make this judgement.
- Detection bias- autopsy procedure: we scored ‘low risk’ when autopsies were standardised, lung examination methods were described, and there was adequate correlation with medical history, ‘high risk’ when there was non-standardised approach to autopsy or inadequate correlation with medical history, and ‘unclear risk’ when there was insufficient information to make this judgement.
- Detection bias- clinical criteria: we scored ‘low risk’ when studies described adequate detail on methods used to classify lung cancer as incidental as opposed to clinically significant, ‘high risk’ when there was no differentiation between subclinical and clinically significant, and ‘unclear risk’ when there was insufficient information to make this judgement.
- Incomplete outcome data: we scored ‘low risk’ when all autopsy cases were included in results, ‘high risk’ when not all cases where included, ‘unclear risk’ when there was insufficient information to make this judgement.
- External validity-generalisability: we scored ‘low risk’ when the study participants were representative of the population, ‘high risk’ when they were not representative, and ‘unclear risk’ when there was insufficient information to make this judgement.
- Other sources of bias: we scored ‘low risk’ when the study did not appear to have other sources of bias, ‘high risk’ when there was at least one other important bias, for example, deviations to protocol, and ‘unclear risk’ when there was insufficient information to make this judgement.

### Analysis

We performed meta-analyses of prevalence using a random intercept logistic regression model in R version 4.2.2 [22], using the packages ‘meta’ version 6.0 [23] and ‘metafor’ version 3.8 [24]. The results of the meta-analyses were presented visually using forest plots. Statistical heterogeneity of prevalence between pooled studies was evaluated using I^2^ statistic and between-study variance with tau^2^ [25].

We performed subgroup analyses for the following;

1. Consecutive versus non-consecutive case selection.
2. Setting – hospital or forensic versus population-based studies.
3. Location – continent.
4. Age – median/mean study age of 40–59 years, 60–89 years, and 90 or greater years.
5. Risk factors.
6. Histological types.
7. Lung cancer stage.

Sex was assessed using mixed-effects model meta-regression analyses.

We performed two sensitivity analyses (1) including high quality studies only, i.e. excluding studies which potentially included people under the age of 18 years old, and (2) including those with low risk of bias autopsy procedures.

Publication bias was assessed using the Luis Furuya-Kanamori asymmetry index (LFK index) and the Doi plot.

## Results

### Results of the search

**Fig. 1:**
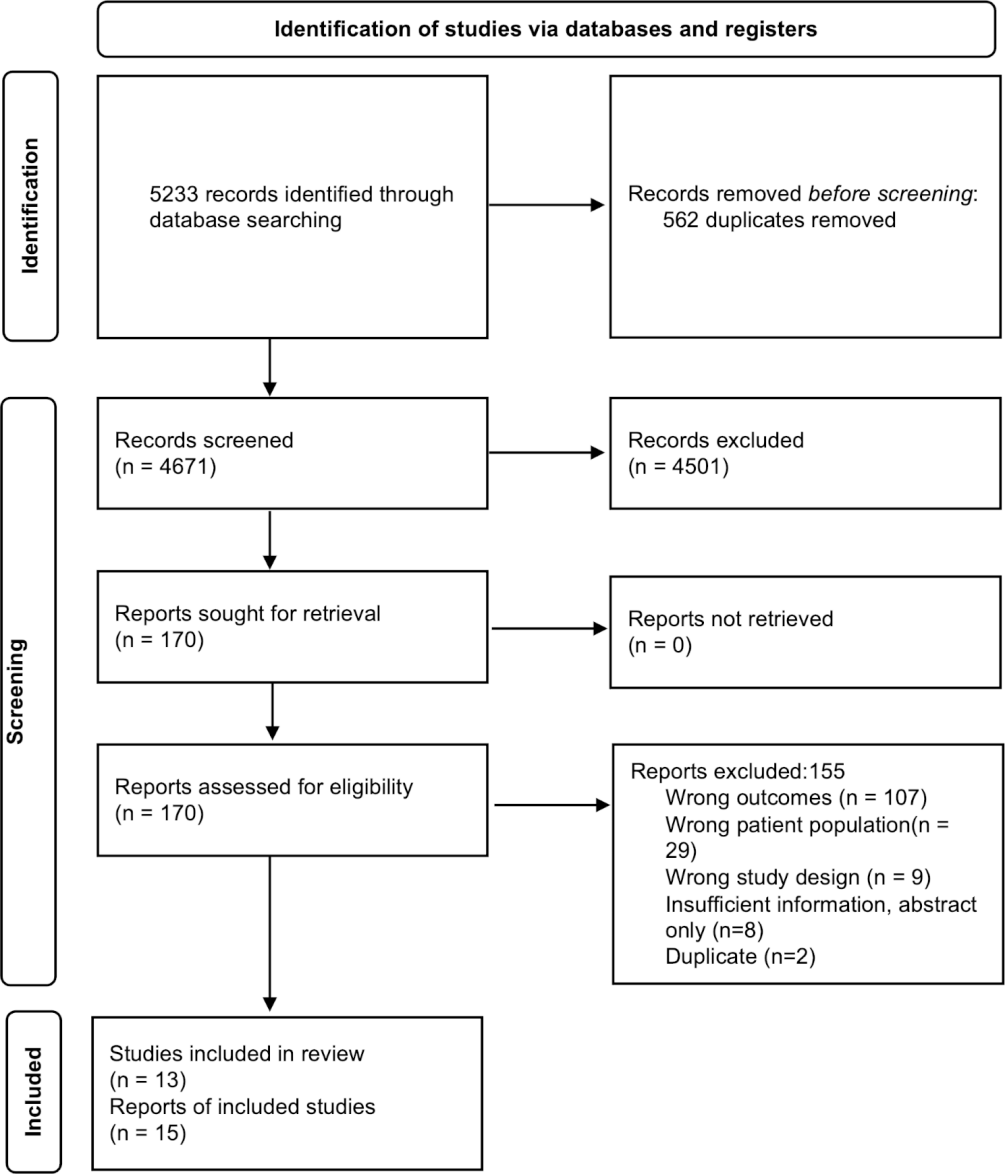
Study selection flow diagram

5233 citations were identified during our database search, of which 170 were selected for full text review. 4501 records were excluded after review of the title and/or abstract as they were judged irrelevant. 13 studies (with a total of 15 citations) with a total of 16 730 autopsies were included in this systematic review. 155 studies did not meet inclusion criteria and were excluded, and reasons are detailed in Fig. 1.

### Included studies

Of the 13 studies, 6 were conducted in North America [26–31], 5 in Europe [16, 32–36], and 2 in Asia [37–39] (Table 1).

**Table 1:** Characteristics of 13 included studies

Study and Year of Publication	Country	Median year when autopsies were performed	Study Population	Number of Autopsies Examined	Consecutive	Median age at Death, range(years)	Female (%)	Sources of funding and Conflicts of Interest (COI)
**Berezowska 2021(32)**	Switzerland	2017	Hospital	189	Yes	69, 26–104	63 (50)	No external funding. No COI declared.
**Burrows 1975(26)**	USA	1973	Hospital	252	NS	NS (inclusion of adults only)	NS	NS
**Gezelius. 1988(16)**	Sweden	1982	Forensic	7020	Yes	Median age not specified. Mean age for men 55 years +/- 20 years SD. Mean age for women 58 years +/- 21 years SD	NS	NS
**Hudak 2022 (33, 34)**	Hungary	1994**	Stroke	534	Yes	Median age not specified. Mean age 70.4 +/- 12.6 years SD (adults only included)**	251 (47)	National Research, Development and Innovation Fund, GINOP-2.3.2-15-2016-00048 (Stay Alive), ELKH-DE Cerebrovascular and Neurodegenerative Research Group. No COI declared.
**Imaida 1997 (37)**	Japan	1988	Hospital	871	NS	Estimated median 82, 48 to 100+	510 (59)	Grant in aid for cancer research from the Ministry of Health and Welfare, Japan. Grant from the Society of Promotion of Toxicologic Pathology. COI NS.
**Ishii 1979 (38, 39)**	Japan	1966	NS	1366	NS	Estimated median 67, 65+	432 (35)	NS
**Murphy 1977 (27)**	USA	1977*	Forensic	1300	Yes	NS	NS	NS
**Rosenblatt 1973 (28)**	USA	1966	Hospital	466	NS	NS	NS	NS
**Sclare 1991(35)**	Scotland	1979* (based on 24-year period and date published)	Hospital	143	No	NS, 90–100 years old	91 (64)	NS
**Sens 2009 (29)**	USA	2009 (date published)*	Majority of cases forensic	412	NS	NS. Mean age 62 years for unsuspected cancers.	155 (38)	NS
**Stanta 1997 (36)**	Italy	1967 (based on 20-year time frame and publication 1997)*	General, older population	267	No	NS, 95–106	214 (80)	NS
**Suen 1974 (30)**	USA	1965	Hospital	3535	Yes	Estimated median 70, 66–107	1693 (48)	NS
**Torbenson 2001 (31)**	USA	1989	Transplant	375	Yes	NS. Mean age 46, SD 11 years	158 (42)	NS

*Estimated based on stated timeframe or year of publication if not otherwise stated

** Confirmed with authors

NS = not specified

Ishii 1979 [38, 39] was the earliest study to commence in 1955, with three other studies having an end date before 1979 [26, 28, 30]. Four studies ended between 1980 and 1999 [16, 31, 33, 37]. The most recent study to conclude was Berezowska 2021 [32] in 2017. Four studies did not specify the specific decade autopsies were conducted (Sclare 1991 [35], Sens 2009 [29], Murphy 1977 [27], Stanta 1997 [36], although Stanta 1997 [36]reported a 20-year period for autopsies, Sens 2009 [29]reported a 5-year period of autopsies, and Sclare 1991 [35] reported a 24-year period for autopsies.

Five studies (Berezowksa 2021 [32], Gezelius 1988 [16], Burrows 1975 [26], Sens [29], Imaida 1997 [37] included all autopsies performed during the study period at their institution. Berezowska 2021 [32] included all adults 18 years and older. Burrows 1975 [26] also included adults only. Two studies, Ishii 1979 [39] and Suen 1974 [30], included people 65 years and older. Sclare 1991 [35] restricted subjects to those aged 90 to 100 years old and Stanta 1997 [36] included only those aged 99 years and older in the analysis. Murphy 1977 [27] comprised of autopsies completed by the author exclusively. Three studies had specific co-morbid cohorts [28, 31, 33]. Hudak 2022 [33] included participants who had a stroke and died. Rosenblatt 1973 [28] included only those who had a malignant disease listed as the cause of death. Torbenson 2001 [31] included patients who had died within 100 days of a solid organ transplant. Four studies did not adequately specify age inclusions to determine children were excluded with certainty [16, 27–29]. Ten studies did not have any specified exclusion criteria [16, 26–28, 30, 32, 33, 35, 36, 39]. Imaida 1997 [37] specified cases with incomplete autopsy records were excluded and Torbenson 2001 [31] specified cases with incomplete autopsies were excluded. Sens 2009 [29] excluded cases of intrauterine foetal demise, skeletal remains, and externally referred neuropathology cases.

Autopsy procedure and thoroughness of examination for included studies are detailed in table S1 (supplementary materials).

Of the 13 studies, five studies (Gezelius 1988, Imaida 1997, Ishii 1979, Sclare 1991 and Santa 1997) had primary objectives focused on malignancy. Four studies (Rosenblatt 1973, Sens 2009, Suen 1974, and Torbenson 2001) had a primary objective focused on subclinical or clinically unsuspected cancer. Three studies (Berezowska 2021, Burrows 1975, and Hudak 2022) primarily focused on diagnostic accuracy and the value of autopsy. One study (Ishii 1979) had no specified objective other than to present autopsy findings in general.

### Excluded studies

We excluded 155 studies. 107 for wrong outcomes, 29 had the wrong patient population, 9 studies were the wrong study design, 8 studies were abstracts only and provided insufficient information to evaluate eligibility for inclusion, and 2 studies were duplicates.

### Risk of bias

**Fig. 2:**
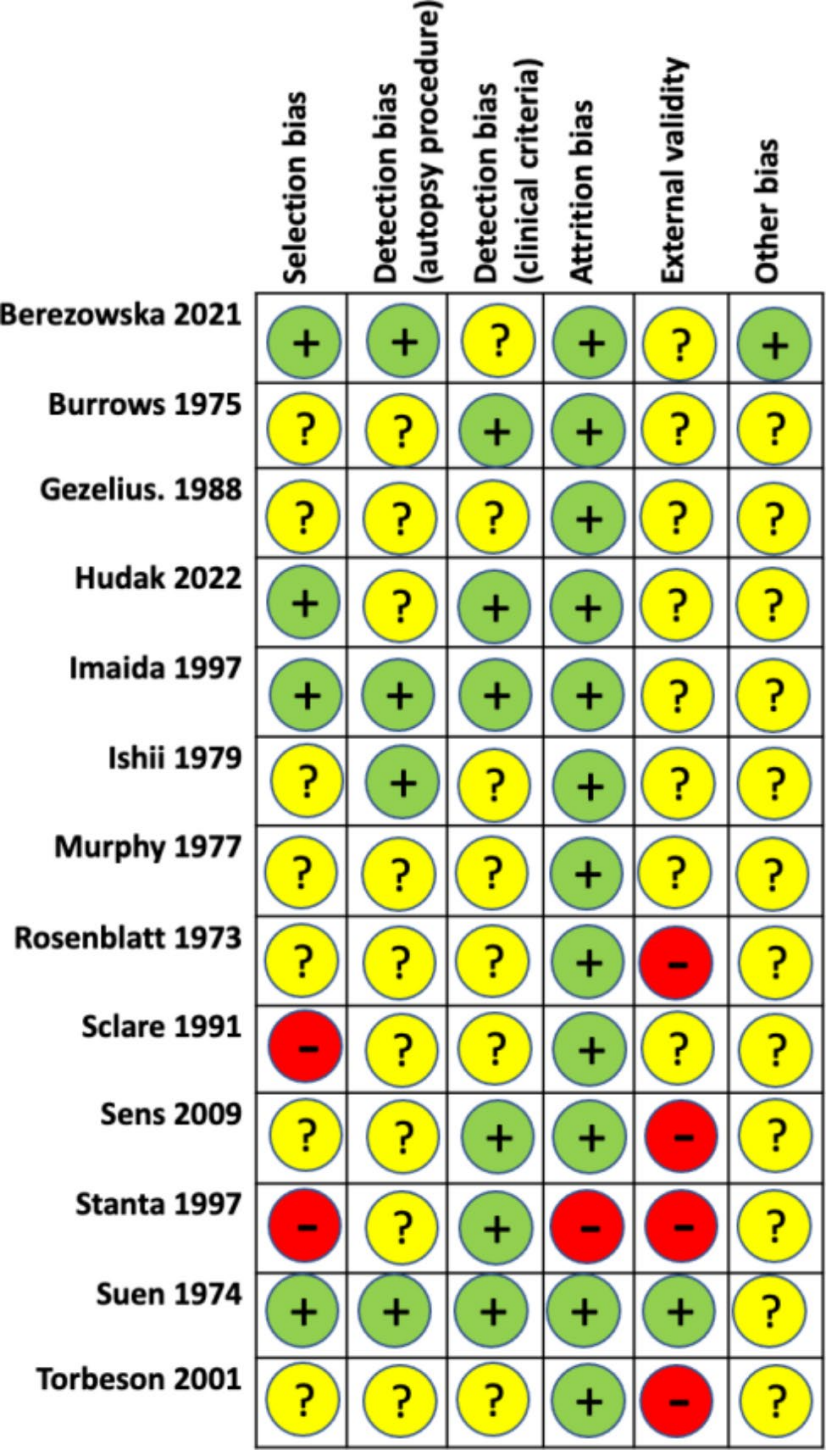
Risk of bias summary

We performed risk of bias assessment for all included studies and summarised the results in Fig. 2. Justifications for grading are detailed in table S2 (supplementary materials).

### Outcomes

Prevalence of lung cancer in the included studies is summarised in Table 2. Given the small number of lung cancers diagnosed antemortem or post-mortem cause of death, the total autopsy denominator was not adjusted to calculate prevalence of subclinical lung cancer as corresponding data were often not available for subgroups and the authors judged the impact to be small.

**Table 2:** Lung cancer prevalence

	Total # of Autopsies Examined	# Subclinical lung cancers diagnosed post-mortem (%)	# Lung cancers diagnosed post-mortem and COD	# Lung cancers diagnosed antemortem
**Gezelius. 1988(16)**	7020	5 (0.07)	12	8
**Suen 1974 (30)**	3535	47 (1.33)	0*	182
**Ishii 1979 (38, 39)**	1366	5 (0.37)	0*	120
**Murphy 1977 (27)**	1300	1 (0.08)	0	2
**Imaida 1997 (37)**	871	23 (2.64)	NS	50
**Hudak 2022 (33, 34)**	534	1 (0.19)	0	NS
**Rosenblatt 1973 (28)**	466	0 (0)	0	27
**Sens 2009 (29)**	412	4 (0.97)	4	1*
**Torbenson 2001 (31)**	375	2 (0.53)	0*	0*
**Stanta 1997 (36)**	267	2 (0.75)	1	0
**Burrows 1975(26)**	252	2 (1.40)	0	NS
**Berezowska 2021(32)**	189	5 (0.07)	NS	16
**Sclare 1991 (35)**	143	47 (1.33)	0	2

NS = not specified, * indicates number calculated from paper

**Fig. 3:**
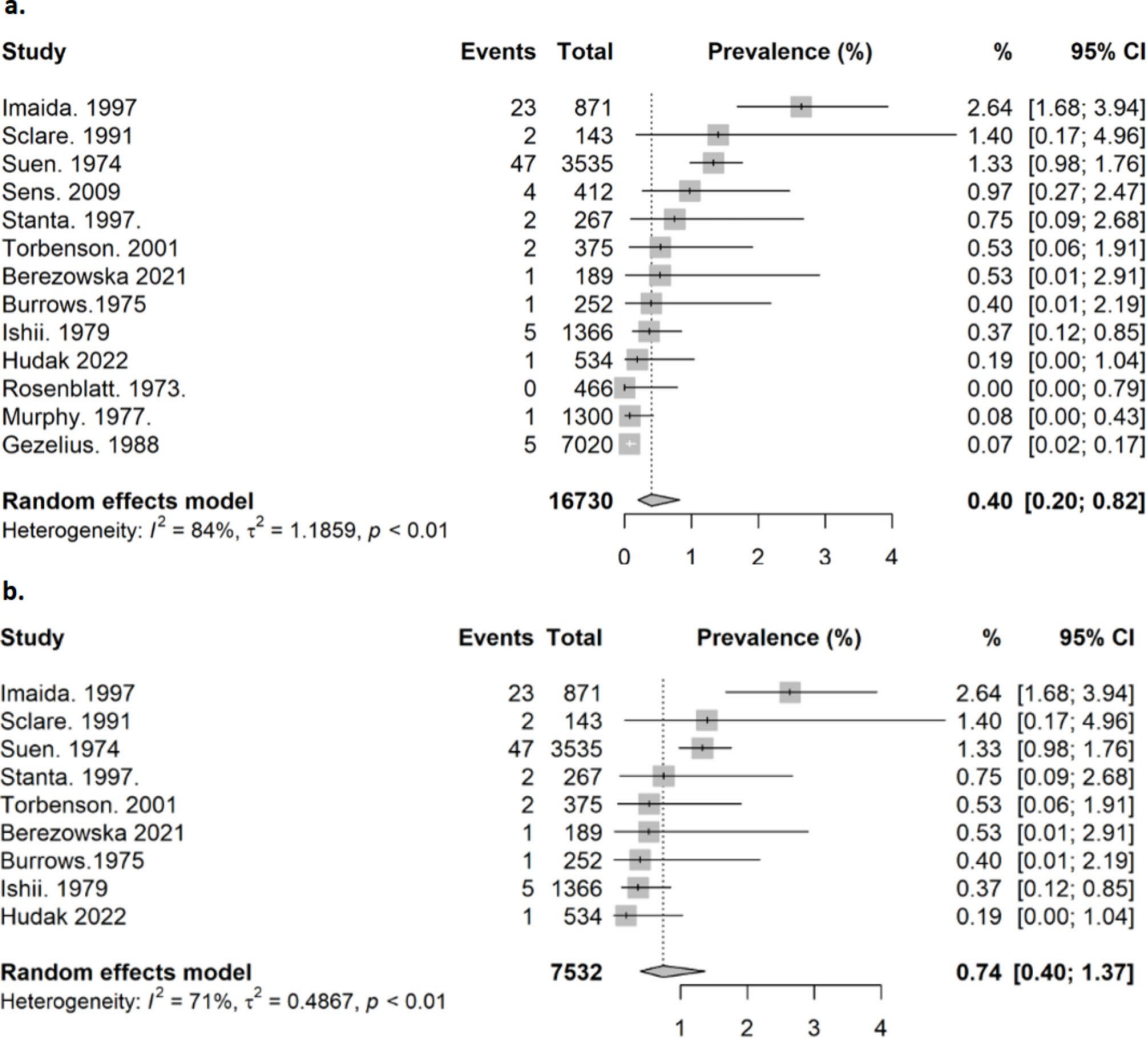
**(a)** Pooled prevalence of subclinical lung cancer (all studies). **(b)** Pooled prevalence of subclinical lung cancer (studies which specified children were not included)

We pooled the prevalence of subclinical lung cancers diagnosed post-mortem for all 13 trials. The evidence showed a pooled prevalence of 0.4% (95% CI 0.20 to 0.82%, I^2^ = 84%, tau^2^ = 1.19; low certainty evidence, 16 730 autopsies, Fig. 3a). Heterogeneity amongst studies was high, with Imaida 1997 [37] having a higher prevalence than the other included studies. Imaida 1997 [37] was conducted in Japan and recruited from a hospital population with a median age of 82 years and the primary objective. We performed a sensitivity analysis excluding studies which did not specify exclusion of children (< 18 years old) in their cohort [16, 27–29]. Nine studies were included in the analysis, with a pooled prevalence of subclinical lung cancer of 0.74% (95% CI 0.40 to 1.37%, I^2^ = 71%, tau^2^ = 0.49, 7532 autopsies, Fig. 3b). Heterogeneity amongst studies was high, however had decreased comparatively.

When we performed another sensitivity analysis of studies with low risk of bias for autopsy procedure. Four studies were included [30, 32, 37, 39], with a pooled prevalence of 1.02% (95% CI 0.45 to 2.20%, I^2^ = 85%, tau^2^ = 0.51, 5961 autopsies).

We performed a meta-regression analysis (proportion male) using the 9 available studies [29–33, 35–37, 39] which showed no statistically significant association with sex, with an odds ratio (OR) = 0.085 (95% CI: 0.003, 2.55; p = 0.155).

We performed the following subgroup analyses.

- By location: All 13 studies were included in this analysis by continent. Analysis provided in supplementary material (Figure S1 supplementary materials).
  1. Asia: Two studies [37, 39] were included in this analysis. Pooled prevalence was 1.01% (95% CI 0.25 to 4.04%, I^2^ = 94%, tau^2^ = 0.93, 2237 autopsies).
  2. Europe: Five studies [16, 32, 33, 35, 36] were included in this analysis. Pooled prevalence was 0.27% (95% CI 0.09 to 0.84%, I^2^ = 76%, tau^2^ = 0.88, 8153 autopsies).
  3. North America: Six studies [26–31] were included in this analysis. Pooled prevalence was 0.37% (95% CI 0.13 to 1.07%, I^2^ = 54%, tau^2^ = 0.98, 6340 autopsies).
  There was no statistically significant difference between subgroups. Test for subgroup differences: Chi^2^ = 2.09, df = 2 (p = 0.35).
- By age: Ten studies provided information regarding age. Analysis provided in supplementary material (Figure S2 supplementary materials).
  1. For those with a median or mean age of 40 to 59 years old [16, 31]: prevalence of subclinical lung cancer was 0.13% (95% CI 0.03 to 0.59%, I^2^ = 83%, tau^2^ = 0.47, 7385 autopsies). Gezelius 1988 [16] did not clearly exclude children, when this study was removed from the analysis, prevalence was 0.52% (91% 0.06 to 1.91%, 1 study [31], 375 autopsies).
  2. For those with a median or mean age of 60 to 89 years old [29, 30, 32, 33, 37, 39]: prevalence of subclinical lung cancer was 0.81% (95% CI 0.0.38 to 1.69%, I^2^ = 79%, tau^2^ = 0.57, 6907 autopsies Sens 2009 [29] did not clearly exclude children, when this study was removed from the analysis, prevalence was 0.76% (95% CI 0.31 to 1.86%, I^2^ = 83%, tau^2^ = 0.0.78, 5 studies, 6495 autopsies).
  3. For those with a median or mean age of ≥90 years old [35, 36]: prevalence of subclinical lung cancer was 0.98% (95% CI 0.37 to 2.57%, I^2^ = 0%, tau^2^ = 0, 410 autopsies). All studies clearly specified they did not include children.
  There was no statistically significant difference between subgroups. Test for subgroup differences: Chi^2^ = 5.29, df = 2 (p = 0.07).

Subgroup analyses by patient selection and setting, study period presented in Table S3, Figure S3, Figure S4, and Figure S5 (supplementary materials). There were no statistically significant differences in subgroup analyses. Pooled analyses for histology were performed separately for cancer subtypes and were also summarised in Table S3 (supplementary materials).

We attempted analysis of studies by stage, however there was insufficient number of studies within each category to proceed. Only three studies reported stages of subclinical lung cancers diagnosed post-mortem [26, 27, 31]. Burrows 1975 [26] reported one case of stage 4 lung cancer. Murphy 1977 [27] and Torbenson 2001 [31] reported 1 and 2 cases of stage 1 lung cancer respectively.

There was inadequate information provided in the studies to perform analyses looking at risk factors including smoking status.

The LFK index was 4.32, with significant asymmetry (Doi plot presented in Figure S6 of the supplementary materials).

## Discussion

Our systematic review of 13 studies of 16 730 autopsies across seven countries and seven decades demonstrated a pooled prevalence of 0.4% for subclinical lung cancer diagnosed post-mortem. When sensitively analysis was performed excluding studies which did not clearly provide age ranges, the pooled prevalence was 0.74%. The LFK index did demonstrate asymmetry, suggestive of possible small study effects, with a larger prevalence estimate and less precision potentially overestimating the prevalence.

To our knowledge this is the first systematic review of the prevalence of subclinical lung cancer detected at autopsies in adults. Strauss 1993 [40] conducted a brief narrative review of subclinical lung cancer diagnosed at autopsy, however the autopsy cases were contaminated with clinically unsuspected lung cancer which was the cause of death. There was one large autopsy study that was excluded from this review as the cohort included children [41]. Karwinski 1990 [41] was Norwegian series of 21, 530 autopsies conducted in people aged 1 to 99 years old. The age range for subclinical lung cancers was 40 to 93 years old and there were 14 cases diagnosed post-mortem (0.65% prevalence), similar to the findings in this review. Our results are also reasonably consistent with the meta-analysis of LDCT lung cancer screening RCTs (in studies with at least 10 years of follow up) which estimated 7 cases of lung cancer overdiagnosis for every 1000 people screened (95% CI of 2 to 84 cases) [4]. It should be noted that the study population in the RCTs were high-risk populations for lung cancer with smoking histories, as opposed to the general population. The largest lung cancer screening with LDCT RCT, the National Lung Screen Trial (NLST), reported at 11.3 year follow-up a lung cancer incidence of 1701 cases in the LDCT screening group (6.3% of their LDCT cohort) [42]. Risk of overdiagnosis is challenging to assess in the NLST given the comparison group received CXR. After 10 years of follow-up, the Dutch–Belgian lung-cancer screening trial (Nederlands–Leuvens Longkanker Screenings Onderzoek [NELSON]) reported a cumulative incidence of 344 lung cancers in their LDCT screening group, data for male participants only provided (5.2% of their male LDCT screening cohort) [43]. The cumulative incidence of lung cancer amongst male participants in NESLON trial control group was 4.6%. With an extended follow-up to 11 years post randomisation, the NELSON study estimated an excess-incidence overdiagnosis rate of 8.9% (95% CI -18.2 to 32.4%) [43]. It is clear screening detects both clinically relevant lung cancers and those which may not progress to cause symptoms or death.

The pooled prevalence described in this review represents the best available estimate of the reservoir of subclinical lung cancer to date and compared to similar reviews in other types of cancer (breast, prostate, and thyroid), was significantly lower [10–12].

The strengths of this review include its comprehensive search strategy, with no language barriers, and thorough evaluation of study methodology. However, there were some limitations. Firstly, this systematic review and search focused on post-mortem diagnoses of lung cancer, and as such studies which evaluated for pre-cursor lesions only were excluded. Two autopsies series which have evaluated precursor adenocarcinoma lesions are Sterner 1997 [44] and Yokose 2000 [45]. Sterner 1997 [44] was a review of 100 consecutive autopsies in the USA and found two cases of atypical alveolar cell hyperplasia in a general autopsy population. Yokose 2000 [45] was an autopsy series of 241 cases in Japan and found 16 people had evidence of atypical adenomatous hyperplasia. Secondly, it should be acknowledged that the background rates of CT in each country during the autopsy period was not readily available. None of the included studies had the primary aim focused on subclinical lung cancer diagnosed at autopsy. In one excluded retrospective study [46] which had the primary aim of detecting subclinical lung cancer at autopsy, 47 cases were found amongst 24. 708 autopsies in a coronial population (0.34% prevalence, 95% CI 0.24 to 0.44%). This study was excluded as the population included children, although the median age was 67 years old.

The evidence in this review is low certainty due to the risk of bias in included studies, possible publication bias and small study effect, and the significant heterogeneity between studies. However, in the case of prevalence studies, heterogeneity may also provide confidence that the outcome is relevant to a wider population and was present in other autopsy reviews [12]. Most studies were published before the year 2000 and descriptions of the population and methodology were limited. Pooled prevalence increased with decade of publication with those published earlier having a lower pooled prevalence. Most study periods were before 1999, with only 601 autopsies being conducted after the year 2000. As such, consideration regarding background smoking rates, use of cigarette filters, environmental exposures should be given. There were concerns listed about the thoroughness of autopsies in some studies which may have underestimated the prevalence of lung cancers, particularly in detecting subsolid or ground glass lesions. The sensitivity analysis in this review including only those studies with a low risk of bias for autopsy procedure had a higher pooled prevalence of subclinical lung cancer, however confidence intervals were overlapping. A previous study in the USA compared 28 patients with post-mortem examinations who had had a CT within 2 months of their death [47]. They found that 19 patients had nodules 15 mm or less in diameter noted on CT and 9 patients had no mention of nodules on autopsy. This may suggest the limitations of autopsy and need for high quality studies, although it could also be the result of interval lesion resolution.

Only five studies reported histology of subclinical lung cancers, with a pooled prevalence of 0.14%, 0.11%, 0.11%, 0.05% for NSCLC not otherwise specified, adenocarcinoma, squamous cell carcinoma (SCC), and bronchoalveolar carcinoma (BAC) respectively. This was a small cohort, with only 1840 autopsies included. Interestingly, in LDCT screening RCTs, whilst at baseline screening SCC, adenocarcinoma and BAC are more common in the LDCT screening compared with the control groups, at later time points, only adenocarcinoma and BAC remain more prevalent in the LDCT cohort [4]. Whilst adenocarcinoma-spectrum lesions growth patterns are more associated with overdiagnoses [9], due to the risk of competing mortality in those with a history of tobacco exposure, there is the potential for all histological types in lung cancer may be overdiagnosed.

Whilst there were no statistically significant differences between the subgroups, there were some trends observed. The pooled prevalence based on patient selection demonstrated a lower prevalence of subclinical lung cancers in studies which recruited consecutively, compared to those which were non-consecutive. Studies from Asia (both studies were conducted in Japan) had the highest pooled prevalence compared to Europe and North America. With both Imaida 1997 and Ishii 1979 also having a low risk of bias for autopsy procedure. This may reflect the importance of race as a risk factor for lung cancer development, particularly low VDT adenocarcinoma, which may contribute to higher risks of overdiagnosis in certain populations [48]. The prevalence also increased with age and is significant as most screening programs are targeting those aged 50 to 80 years old.

In order to advance our understanding about the risk of overdiagnosis with lung cancer screening and the natural history of lung cancer, more information is needed. Further research is required regarding the reservoir of precursor lung lesions and high-quality, prospective studies assessing post-mortem diagnosis of subclinical lung cancer in adult populations, including those who would be eligible for lung cancer screening.

## Conclusions

This review has found that the reservoir for subclinical lung cancer is relatively small in published autopsy series, suggesting a relatively low risk of overdiagnosis with screening compared with breast, prostate and thyroid cancer. .

### Electronic supplementary material

Below is the link to the electronic supplementary material.

Supplementary Material 1

## Data Availability

All data analysed during this review are included in this published article and its supplementary information files.

## List of abbreviations

LDCT: Low-dose computed tomography
LFK: Luis Furuya-Kanamori asymmetry index
PRISMA: Preferred Reporting Items for Systematic Reviews and Meta-analyses
RCT: Randomised controlled trial
RoB: Risk of bias
SCLC: Small cell lung cancer
USA: United States of America
VDT: Volume doubling time
WHO: World Health Organization
