# Supplementary material for: Prevalence of subclinical lung cancer detected at autopsy: a systematic review
Source: BMC Cancer. 2023 Aug 24;23:794. doi: 10.1186/s12885-023-11224-3 (PMC10463584; doi:10.1186/s12885-023-11224-3)

**Table S1. Autopsy Examination**

| **Study and Year of Publication** | **Autopsy Protocol** |
| --- | --- |
| **Berezowska 2021(29)** | Standardised protocol. Both lungs were removed after evisceration of the heart and evaluation of the large pulmonary arteries for central pulmonary emboli. Four percent buffered formaldehyde was instilled through a large bronchi using a large 60mL syringe. After fixation for a least 24 hours, large frontal tissue sections of 1-2 cm thickness were prepared using a support device tailor-made for the purpose of lung sectioning. After sectioning, the parenchyma was carefully evaluated. Standardised histology samples were taken from every lobe and from macroscopically abnormal areas, if identified. Histological stains comprised hematoxylin-eosin for all samples and additional Periodic acid Schiff (PAS) reaction, Elastica van Gieson (EvG) and Iron stain on at least one tissue block. Frocott's methenamine silver (GMS) staining was only performed in cases with suspicion on fungal infection if PAS staining was negative |
| **Burrows 1975(23)** | Autopsies performed according to protocol (not specified) by American Board of Pathology certified pathologists, except for approximately 20 autopsies which were performed by a resident in pathology under supervision. |
| **Gezelius 1988(13)** | Not specified. |
| **Hudak 2022 (30, 31)** | Autopsies performed according to protocol (not specified), however reported to be consistent with latest international standards. Autopsies consisted of thoracic, abdominal, and pelvic organs, body, and brain autopsy. Specimens were sliced from the brain after 1 week of formalin fixation |
| **Imaida 1997 (34)** | All cases underwent a full autopsy with all internal organs including the brain and spinal cord examined. Specimens were fixed in 10% buffered formalin. Lungs are injected with 10% formalin and then post fixation, each lobe cut into 5-6 slices. |
| **Ishii 1979 (35, 36)** | After macroscopic exam, 30-40 histologic sections were obtained from every available organ of each patient. Microscopic findings were checked by all pathologists in a departmental meeting. |
| **Murphy 1977 (24)** | Autopsy protocol not specified. 1287 autopsies were complete (included examination of brain and all organs of neck and often the cervical spinal cord), 13 were only partial examinations. Neoplasms were reported to be evaluated both grossly and microscopically. |
| **Rosenblatt 1973 (25)** | Not specified. |
| **Sclare 1991 (32)** | Not specified. |
| **Sens 2009 (26)** | Autopsy protocol not specified, reported similar in all cases. |
| **Stanta 1997 (33)** | Not specified. |
| **Suen 1974 (27)** | All autopsies were performed by residents under supervision by pathologists. All tissues, including the cranial contents where permitted, and spinal column and cord, were carefully examined both grossly and histologically. |
| **Torbenson 2001 (28)** | Autopsies were performed using the en masse (Rokitansky) method with sections examined from all organ systems with additional sections examined depending on clinical history and anatomic findings. |

**Table S2. Risk of bias summary: review authors; judgements about each risk of bias item for each included study.**

|  | **Selection bias** | **Detection bias- autopsy procedure** | **Detection bias- clinical criteria** | **Attrition bias** | **External validity** | **Other sources of bias** |
| --- | --- | --- | --- | --- | --- | --- |
| **Berezowska 2021(29)** | Low risk: consecutive cases were selected and children were excluded from the study. | Low risk: autopsy procedure was standardised and assessment was detailed. | Unclear risk: insufficient detail provided, although authors reported liaising with clinicians in many cases. | Low risk: all cases were included in the analysis. | Unclear risk: 32 cases were from the intensive care unit, 34 cases from other hospital departments, 119 cases from the community hospitals and 4 cases from general practitioners or families. | Low risk: no deviation noted. |
| **Burrows 1975(23)** | Unclear risk: authors did not specify if cases were enrolled consecutively, children were not included. | Unclear risk: autopsy protocol not specified. | Low risk: authors specified lung cancer confirmed not known antemortem and not cause of death. | Low risk: all cases were included in the analysis. | Unclear risk: cohort was a large teaching hospital, however demographic information on autopsy cases was not provided. | Unclear risk: insufficient information provided. |
| **Gezelius 1988(13)** | Unclear risk: authors enrolled consecutive cases, however did not provide a clear age range, only a standard deviation, to adequately exclude inclusion of children. | Unclear risk: autopsy protocol not specified. | Unclear risk: insufficient information provided about method of determining antemortem diagnoses. | Low risk: all cases were included in the analysis. | Unclear risk: forensic population, insufficient information provided. | Unclear risk: insufficient information provided. |
| **Hudak 2022 (30, 31)** | Low risk: consecutive cases were enrolled, no children included (confirmed by authors) | Unclear risk: autopsy protocol not specified. Authors reported autopsies were consistent with latest international standards. | Low risk: authors used previously described methods for clinical and autopsy diagnoses (37). | Low risk: all cases were included in the analysis. | Unclear risk: cohort with acute stroke. | Unclear risk: insufficient information provided. |
| **Imaida 1997 (34)** | Low risk: no reports of inappropriate exclusions, adult cohort. | Low risk: autopsy procedure was standardised and assessment was detailed. | Low risk: provided detail on subclinical versus cause of death post mortem diagnoses. | Low risk: all cases were included in the analysis. | Unclear risk: cohort was a hospital population, limited information provided. | Unclear risk: insufficient information provided. |
| **Ishii 1979 (35, 36)** | Unclear risk: limited information provided, children not included. | Low risk: autopsy procedure was standardised and assessment was detailed. | Unclear risk: insufficient information provided. | Low risk: all cases were included in the analysis | Unclear risk: limited data provided. | Unclear risk: insufficient information provided. |
| **Murphy 1977 (24)** | Unclear risk: consecutive cases were enrolled, however limited information provided regarding age. | Unclear risk: autopsy protocol not specified. | Unclear risk: clinical criteria used to define cancers as incidental not provided. | Low risk: all cases were included in the analysis | Unclear risk: forensic population, limited information provided. | Unclear risk: insufficient information provided. |
| **Rosenblatt 1973 (25)** | Unclear risk: authors did not specify if cases were enrolled consecutively, limited information provided regarding age. | Unclear risk: autopsy protocol not specified. | Unclear risk: limited information provided on how clinical information was obtained. | Low risk: all cases were included in the analysis | High risk: all cases had a cancer history. | Unclear risk: insufficient information provided. |
| **Sclare 1991 (32)** | High risk: cases were a random sample from physician. | Unclear risk: autopsy protocol not specified. | Unclear risk: limited information provided, reports correlated with history from the hospital. | Low risk: all cases were included in the analysis. | Unclear risk: limited information provided, older population, cases selected for clinical interest. | Unclear risk: insufficient information provided. |
| **Sens 2009 (26)** | Unclear risk: limited information provided regarding age, authors also excluded 52 cases where there was limitations with the autopsy. | Unclear risk: autopsy protocol not specified, however reported similar in all cases. | Low risk: adequately defined. | Low risk: all cases were included in the analysis. | High risk: unbalanced cohort. 87% of cohort was White, 6% Native American, 1% African American, <1% Asian, 3% mixed racial identification, 4% without racial information, 2% Hispanic, and more males than females were included. | Unclear risk: insufficient information provided. |
| **Stanta 1997 (33)** | High risk: random samples taken for some cases. | Unclear risk: autopsy protocol not specified. | Low risk: adequately defined. | High risk: authors did not include autopsies of cases aged 75 to 90 years old. | High risk: older population, with more females than males, however represented general population of region. | Unclear risk: insufficient information provided. |
| **Suen 1974 (27)** | Low risk: consecutive cases without children included. Excluded 37 cases with limited autopsies, however deemed not significant exclusion. | Low risk: standardised autopsies performed with assessment detailed. All lung lobes had a histologic section. | Low risk: adequately defined. | Low risk: all cases were included in the analysis. | Low risk: hospital cohort. | Unclear risk: insufficient information provided. |
| **Torbenson 2001 (28)** | Unclear risk: transplant cases were consecutively enrolled, however 52 exclusions due to autopsy concerns and limited information provided. | Unclear risk: autopsies were performed using the Rokitansky method, with additional sections examined depending on clinical history. | Unclear risk: limited information provided on how clinical information was obtained. | Low risk: all cases were included in the analysis. | High risk: specific population (transplant recipients), 93% of cohort Caucasian. | Unclear risk: insufficient information provided. |

**Table S3. Summary of Subgroup Analyses.**

| Patient selection (consecutive vs non-consecutive). Figure S3 supplementary materials. | 1. Consecutive: Six studies were included in this analysis(13, 24, 27-30). Pooled prevalence was 0.25% (95% CI 0.09 to 0.72%, I^2^= 90%, tau^2^=1.21, 12 953 autopsies). 2. Non-consecutive. Four studies were included in this analysis (32-34, 36). Pooled prevalence was 0.93% (95% CI 0.40 to 2.39%, I^2^= 83%, tau^2^=0.56, 2647 autopsies).   There was no statistically significant difference between subgroups. Test for subgroup differences: Chi^2^ = 3.79, df = 1 (p = 0.05). |
| --- | --- |
| Setting. Figure S4 supplementary materials | - 1. Hospital: Six studies were included in this analysis(23, 25, 27, 29, 32, 34). Pooled prevalence was 0.80% (95% CI 0.30 to 2.09%, I^2^= 53%, tau^2^=0.78, 5456 autopsies).   2. General (elderly) population: One study was included in this group(33). Prevalence was 0.75% (95% CI 0.09 to 2.68%, 267 autopsies).   3. Forensic/Coronial: Three studies were included in this analysis (13, 24, 26). Pooled prevalence was 0.16% (95% CI 0.04 to 0.68%, I^2^= 88%, tau^2^=1.22, 8732 autopsies).   4. Stroke: One study was included in this group(30). Prevalence was 0.19% (95% CI 0.00 to 1.04%, 534 autopsies).   5. Transplant: One study was included in this group(28). Prevalence was 0.53% (95% CI 0.06 to 1.91%, 375 autopsies).   There was no statistically significant difference between subgroups. Test for subgroup differences: Chi^2^=4.56, df = 4 (p = 0.33). |
| Study period. Figure S5 supplementary materials. | When study period was not clearly defined, date of publication was used as per Table 1.   1. For studies conducted during 1960 to 1979: Five studies(23-25, 27, 36) were included with a pooled prevalence of 0.28% (95% CI 0.08 to 0.96%, I^2^= 75%, tau^2^=1.17, 6919 autopsies). 2. For studies conducted during 1980 to 1999: Six studies were included(13, 28, 30, 32-34), with a pooled prevalence of 0.47% (95% CI 0.15 to 1.41%, I^2^=92%, tau^2^=1.49, 9219 autopsies). 3. For studies conducted during 2000 to 2019: Two studies (26, 29) were included, with a pooled prevalence of 0.83% (95% CI 0.35 to 1.98%, I^2^=0%, tau^2^=0, 601 autopsies).   There was no statistically significant difference between subgroups. Test for subgroup differences: Chi^2^ = 2.11, df = 2 (p = 0.35). |
| Histology (analysis not shown). | Five studies reported histology for all subclinical lung cancers diagnosed (23, 26, 28, 30, 33). Histology is grouped as described by the study, and as such bronchoalveolar carcinoma (BAC) is presented as separate to adenocarcinoma in this review, although BAC was reclassified as various adenocarcinoma subtypes in the most recent WHO classification (8). None of the five studies included reported small cell carcinoma.   1. Adenocarcinoma: pooled prevalence of subclinical adenocarcinoma was 0.11% (95% CI 0.03 to 0.43%, I^2^=0%, tau^2^=<0.0001, 1840 autopsies). 2. BAC: pooled prevalence of subclinical BAC was 0.05% (95% CI 0.01 to 0.38%, I^2^=0%, tau^2^=0, 1840 autopsies). 3. Squamous cell carcinoma (SCC): pooled prevalence of subclinical SCC was 0.11% (95% CI 0.03 to 0.43%, I^2^=0%, tau^2^=0, 1840 autopsies). 4. Adenosquamous cell carcinoma: pooled prevalence of subclinical adenosquamous carcinoma was 0.05% (95% CI 0.01 to 0.38%, I^2^=0%, tau^2^=0, 1840 autopsies). 5. Non-small cell lung carcinoma not otherwise specified (NSCLC NOS): pooled prevalence of NSCLC NOS was 0.14% (95% CI 0.01 to 1%, I^2^=0%, tau^2^= 0.99,1840 autopsies) |

**Figure S1. Pooled prevalence of subclinical lung cancer by geographical location.**


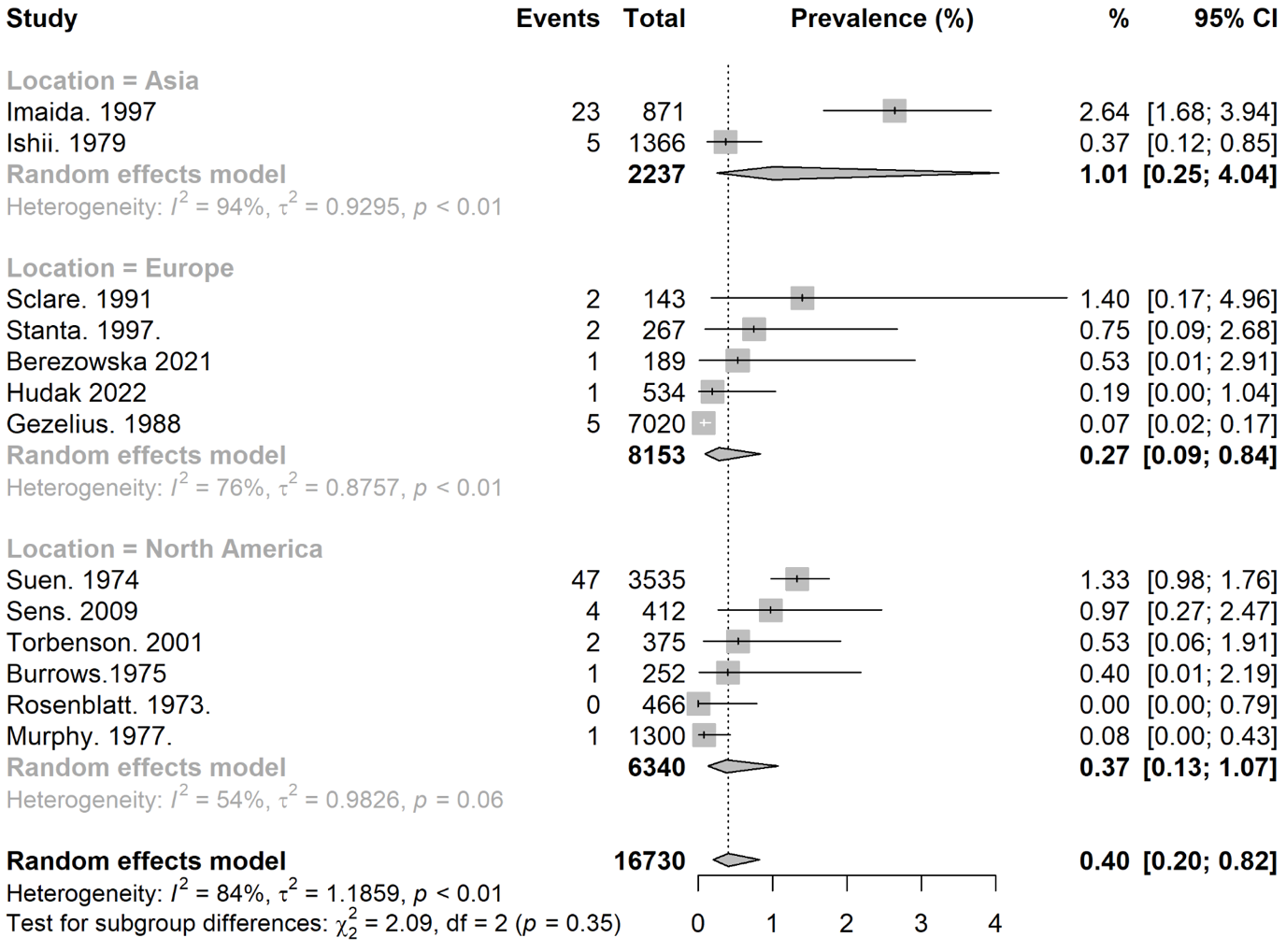


**Figure S2. Pooled prevalence of subclinical lung cancer by age.**


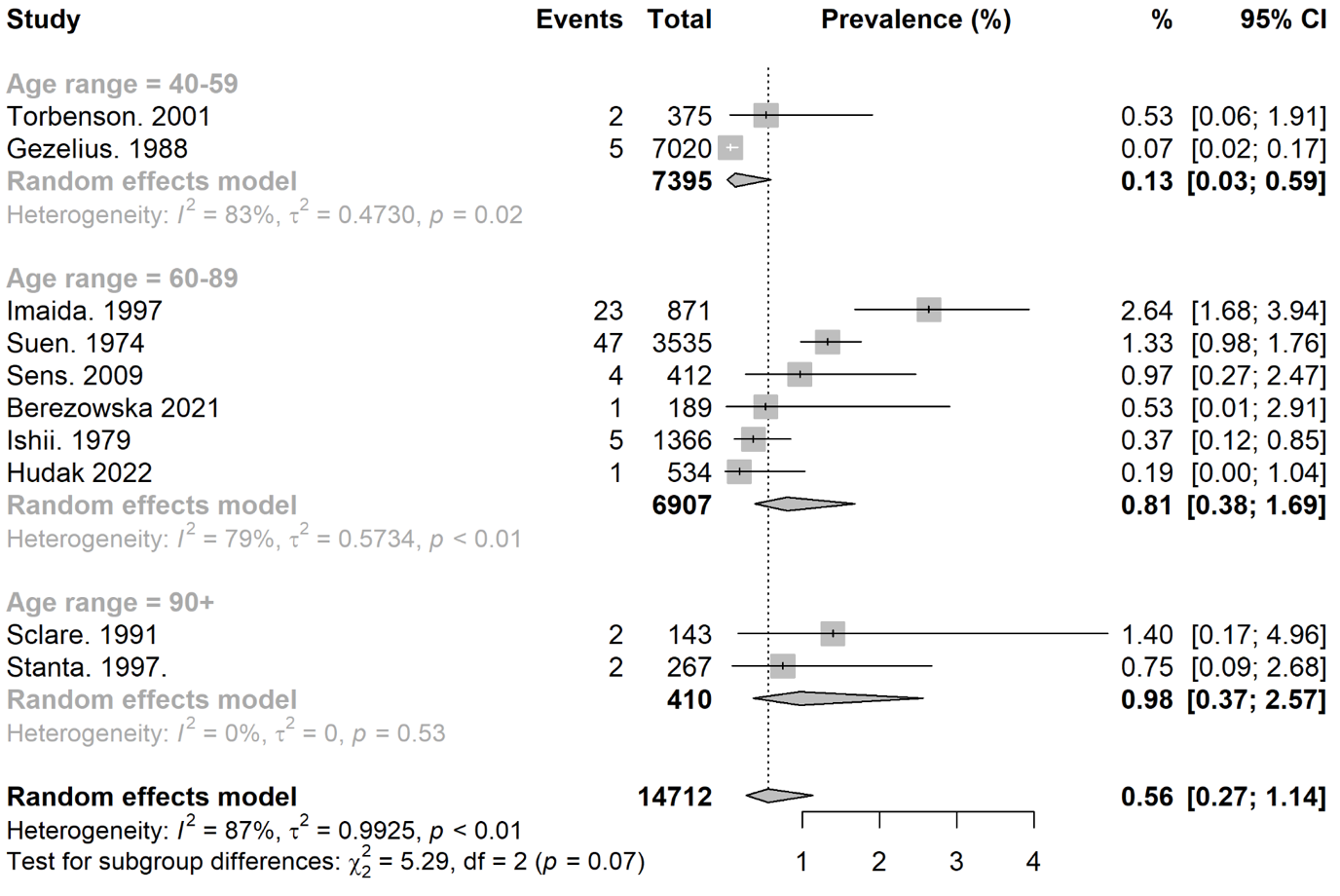


**Figure S3 Pooled prevalence of subclinical lung cancer by patient selection (consecutive vs non-consecutive).**


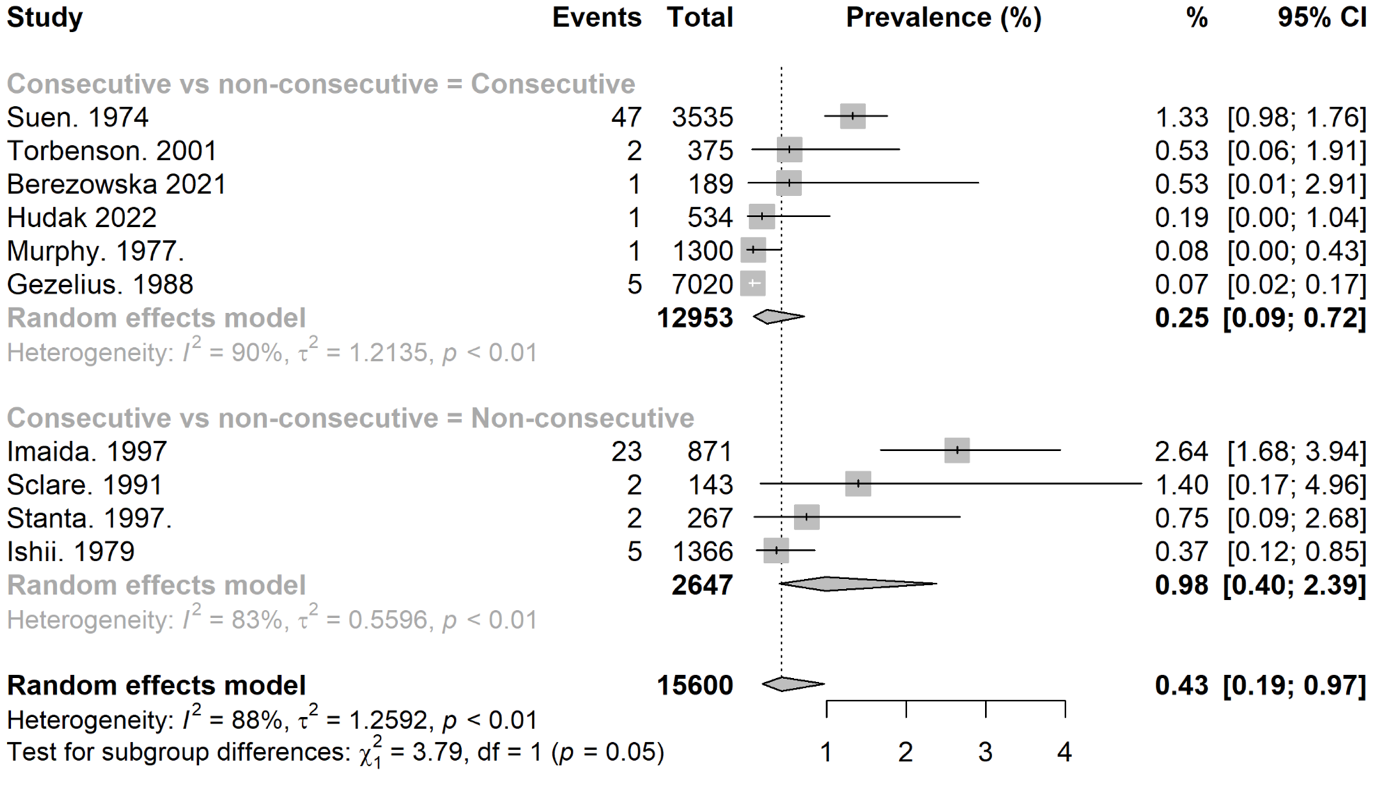


**Figure S4. Pooled prevalence of subclinical lung cancer by setting.**


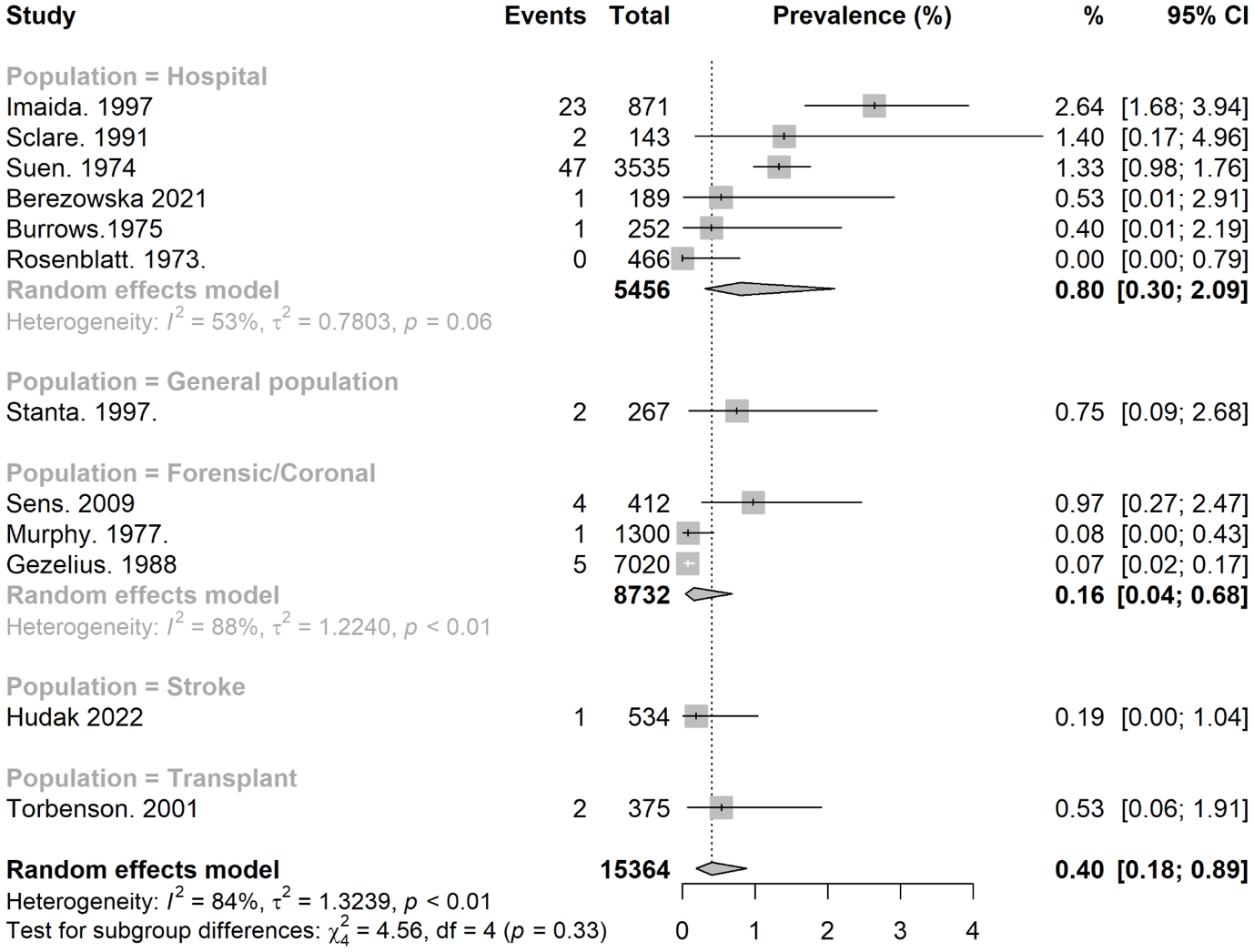


**Figure S5. Pooled prevalence of subclinical lung cancer by study period.
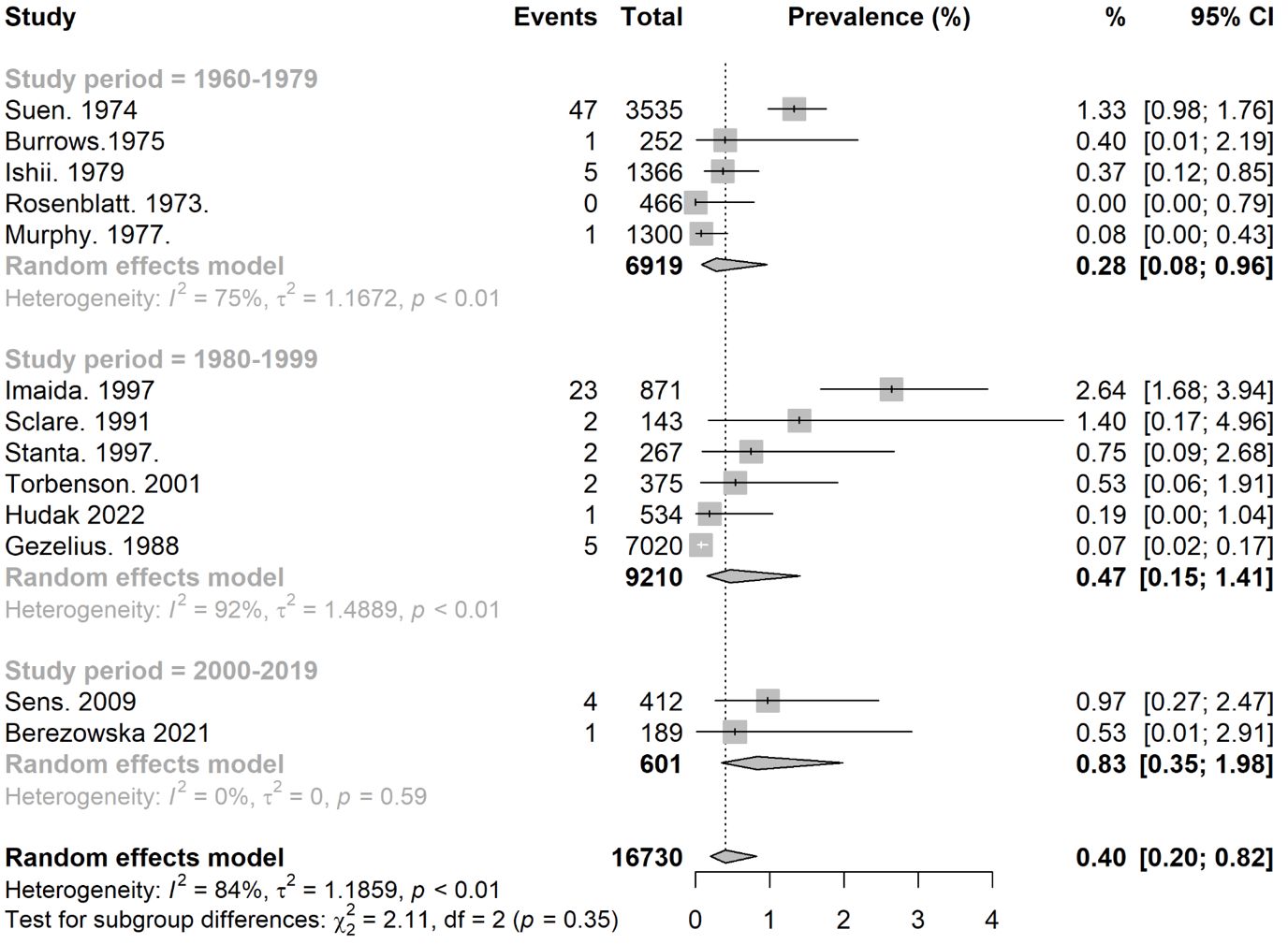
**

**Figure S6. Doi plot of included studies.**


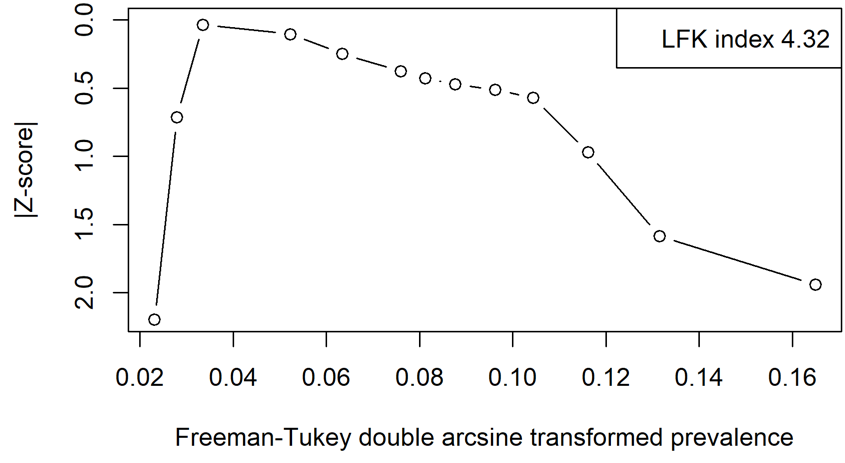

Supplement: Supplementary file 1 — Supplementary Material 1 [file 12885_2023_11224_MOESM1_ESM.docx]
